# Knockdown of the *ABCG23* Gene Disrupts the Development and Lipid Accumulation of *Panonychus citri* (Acari/Tetranychidae)

**DOI:** 10.3390/ijms25020827

**Published:** 2024-01-09

**Authors:** Hongyan Wang, Haifeng Wang, Tianrong Xin, Bin Xia

**Affiliations:** School of Life Sciences, Nanchang University, Nanchang 330031, China; 355600210021@email.ncu.edu.cn (H.W.); 405600220108@email.ncu.edu.cn (H.W.); xintianrong@ncu.edu.cn (T.X.)

**Keywords:** pest management, RNAi, lipid transportation, fecundity

## Abstract

*Panonychus citri* is a worldwide citrus pest that is currently controlled through the use of insecticides. However, alternative strategies are required to manage *P. citri*. Recent studies suggest that the ATP-binding cassette (ABC) transporter G subfamily plays a crucial role in transporting cuticular lipids, which are essential for the insect’s barrier function against microbial penetration. Therefore, investigating the potential of the ABC transporter G subfamily as a control measure for *P. citri* could be a promising approach. Based on the genome database, the gene was cloned, and the transcriptional response of *ABCG23* for the different developmental stages of *P. citri* and under spirobudiclofen stress was investigated. Our results showed that the expression level of *ABCG23* was significantly lower in adult females exposed to treatment compared to the control and was higher in females than males. The knockdown of *ABCG23* using RNAi led to a decrease in the survival rate, fecundity, and TG contents of *P. citri*. Additionally, a lethal phenotype was characterized by body wrinkling and darkening. These results indicate that *ABCG23* may be involved in cuticular lipid transportation and have adverse effects on the development and reproduction of *P. citri*, providing insight into the discovery of new targets for pest management based on the insect cuticle’s penetration barrier function.

## 1. Introduction

ATP-binding cassette (ABC) transporters are the largest transport family found in plants, animals, and other living organisms. They use the energy released by ATP hydrolysis to move a broad range of biological molecules across cell membranes, such as lipids, amino acids, polypeptides, toxins, and chemical drugs [1]. These transporters can be classified into eight subfamilies (*ABCA* to *ABCH*) based on their sequence similarity and domain conservation [2]. ABC transporter proteins consist of the following two domains that are conserved across species: nucleotide-binding domains (NBDs) and transmembrane domains (TMDs).

These domains are crucial for the function of transporter proteins [3,4,5]. In insects, ABC transporters play crucial roles in various physiological processes, including development, reproduction, and insecticide resistance. For instance, when *ApABCG4* was silenced during wing development, approximately 50% of the aphids had malformed wings [6]. The injection of *TcABCH-9C* dsRNA significantly decreased the number of eggs laid by *Tribolium castaneum*, resulting in no egg hatching [7]. Furthermore, the overexpression of *ABCC1* has been associated with resistance against imidacloprid and chlorpyrifos in *Rhopalosiphum padi* [8], while specific ABCA and ABCC genes have been discovered to confer resistance to diflubenzuron in *T. castaneum* [9]. Moreover, ABC transporters have been implicated in the formation of the cuticle barrier in insects [10]. Overall, ABC transporters exhibit a diverse range of functions in insects.

The ABC transporter G subfamily has been identified as the largest subfamily of the ABC transporter across multiple species [11,12]. Recently, the involvement of ABCG transporters in insect development, reproduction, and insecticide resistance has been widely reported. The ABC transporter G subfamily consists of half transporters that require dimerization to form functional homo- or heterodimeric transporters. In insect genomes, a range of 12 to 23 genes from the ABC transporter G subfamily have been identified [13,14,15]. These proteins not only potentially play a role in insecticide elimination but also participate in the transport of cuticular lipids [7,10,16], eye pigment precursors [7,17,18], and ecdysteroids [7,19].

The insect cuticle comprises mainly chitin, proteins, and lipids that interact to form a layered structure [20]. Secreted by the underlying epidermal cells, it covers the surface of the insect body and serves as a muscle attachment site that is necessary for locomotion, maintains the shape of the insect body, prevents water loss, and resists microbial penetration [21]. The loss or alteration of its components can affect the function of cuticles in insects, leading to abnormal growth and development. Therefore, disrupting the formation and function of the cuticle is a promising strategy for controlling pests [10]. The barrier function of the cuticle, which protects against microbial penetration, is primarily determined by the cuticular lipids.

*Panonychus citri* McGregor (Acari/Tetranychidae) is a highly destructive agricultural pest that is commonly controlled through the use of chemical insecticides [22,23,24]. One of these insecticides is spirobudiclofen, a tetronic acid derivative, acaricide, that targets lipid biosynthesis. The toxic stress treatments of the present study refer to our previous study [19] for the experimental results of the toxicity of spirobudiclofen against *P. citri* (LC_30_: 2.945 g/L and LC_50_: 5.847 g/L) [25]. However, to minimize the use of these chemicals, it is necessary to find new methods for managing *P. citri*.

In our study, the *ABCG23* of *P. citri* was identified, which may offer a potential new target gene for controlling these pests. Meanwhile, *PcABCG23* is specifically expressed in adult females and likely plays a role in lipid accumulation. In addition, our study found that inhibiting the expression of *PcABCG23* resulted in decreased survival rates and fecundity in *P. citri*. These findings contribute to our understanding of the molecular functions of *ABCG23* in the transport of insect cuticular lipids. Moreover, these results may help in the discovery of new targets for pest management based on the penetration barrier function.

## 2. Results

### 2.1. Cloning and Sequence Analysis

**Figure 1:**
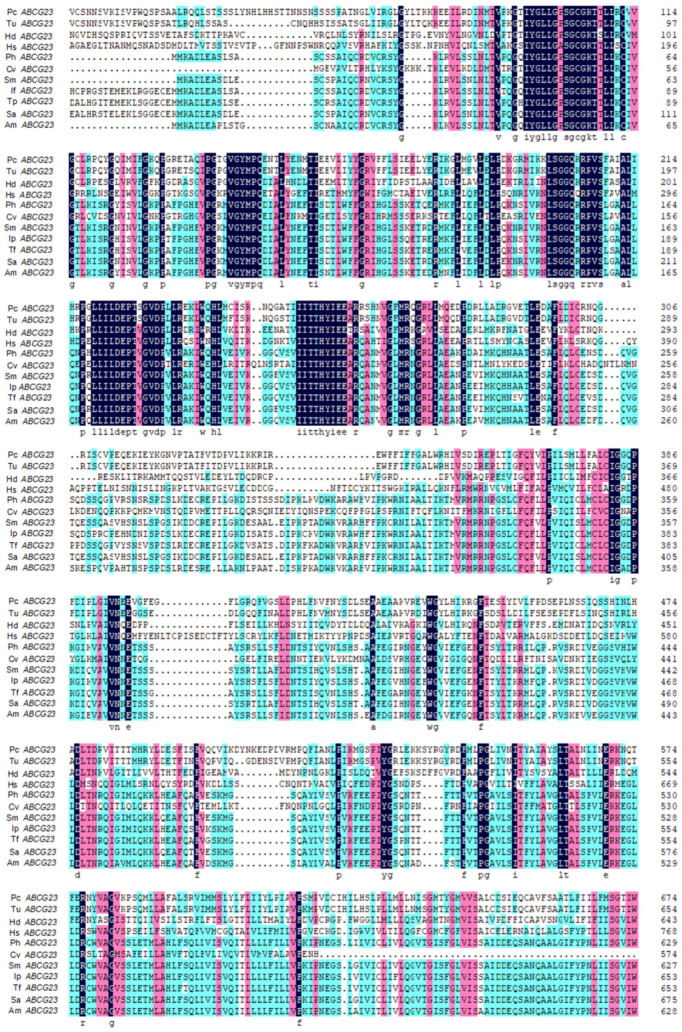
Multiple sequence alignments of *PcABCG23*. The deduced amino acid sequences of *ABCG23* from *P. citri* (OR912982), *T. urticae* (XP_015781176.1), *Halotydeus destructor* (KAI1291018.1), *Harpegnathos saltator* (XP_019699572.1), *Pangasianodon hypophthalmus* (XP_026799872.1), *Crassostrea virginica* (XP_022323233.1), *Silurus meridionalis* (XM_015519618.1), *Ictalurus punctatus* (XP_017331852.1), *Tachysurus fulvidraco* (XP_026998856.1), *Silurus asotus* (XM_001942896.5), and *Astyanax mexicanus* (XP_007244688.1). For the interpretation of the references to color in this figure legend, the reader is referred to the web version of this article.

The cDNA full-length *ABCG23* gene was 2749 bp, including an ORF of 2073 bp that encoded 690 amino acid proteins (GenBank OR912982). The study analyzed multiple alignments of known amino acid sequences of the *ABCG23* transporter gene and found that the sequences were highly conserved among different species, as shown in Figure 1. The amino acid sequence of *PcABCG23* had the highest degree of conservation among acarina, sharing an 82.57% sequence identity with *Tetranychus urticae*.

**Figure 2:**
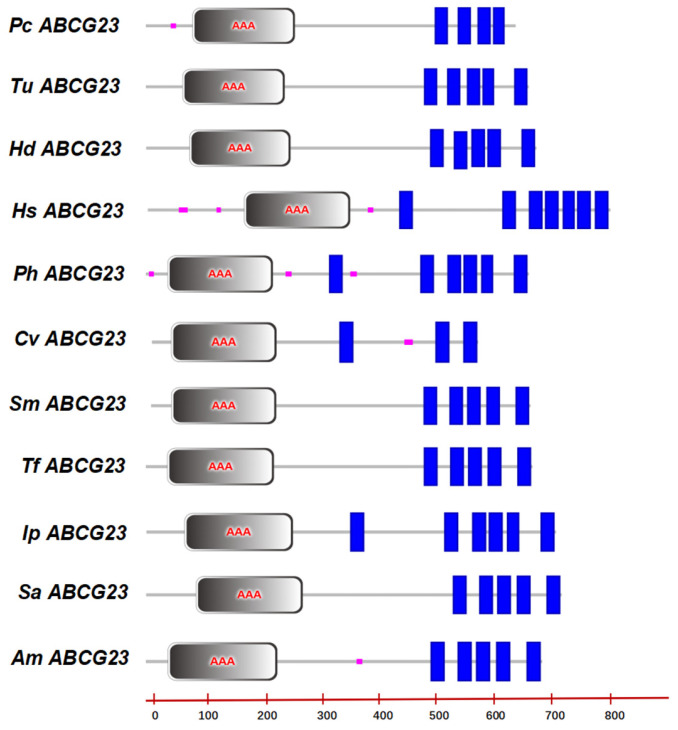
Conserved domain analysis of *P. citri* (OR912982), *T. urticae* (XP_015781176.1), *H. destructor* (KAI1291018.1), *H. saltator* (XP_019699572.1), *P. hypophthalmus* (XP_026799872.1), *C. virginica* (XP_022323233.1), *S. meridionalis* (XM_015519618.1), *T. fulvidraco* (XP_026998856.1), *I. punctatus* (XP_017331852.1), *S. asotus* (XM_001942896.5), and *A. mexicanus* (XP_007244688.1) *ABCG23* transporters. Analysis using SMART online software (SMART: Main page (embl.de)); purple marks signify low complexity; blue marks signify the transmembrane region. The black annular regions with AAA represent NBDs.

SMART online software (https://smart.embl.de/smart/set_mode.cgi?NORMAL=1 accessed on 13 August 2022) analysis revealed that the *PcABCG23* contained 3-7 transmembrane regions. The 11 *ABCG23* genes contained AAA domains (nucleotide binding domains (NBDs), Figure 2). To verify whether there was an error in the *PcABCG23* protein sequence, we analyzed similar sequences from *T. urticae*, *H. destructor*, *H. saltator*, *P. hypophthalmus*, *C. virginica*, *S. meridionalis*, *T. fulvidraco*, *I. punctatus*, *S. asotus*, and *A. mexicanus*. We found that the *ABCG23* gene in other species had the same AAA domain (Figure 2), which indicates that this protein is a member of the ABCG subfamily.

**Figure 3:**
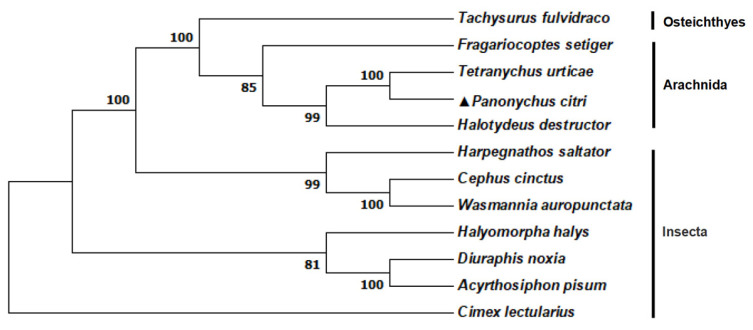
Phylogeny of the *ABCG23* gene. The phylogenetic tree was built based on the amino acid sequences (*Tachysurus fulvidraco* (XP_026998856.1), *Fragariocoptes setiger* (KAG9508995.1), *T. urticae* (XP_015781176.1), *P. citri* (OR912982), *H. destrutor* (KAI1291018.1), *H. saltator* (XP_019699572.1), *Cephus cinctus* (XP_015609354.1), *Wasmannia auropunctata* (XP_011700766.1), *Halyomorpha halys* (XP_014283071.1), *Diuraphis noxia* (XP_015375104.1), *Acyrthosiphon pisum* (XP_001942931.1), and *Cimex lectularius* (XP_014247326.1)) via the Neighbor-Joining method using the program MEGA 7.0, while the phylogenetic test was carried out using a bootstrap analysis with 1000 replications.

Phylogenetic tree analysis revealed a close evolutionary relationship between *PcABCG23* and *TuABCG23*, indicating their recent differentiation and potential functional similarities (refer to Figure 3). In contrast, insects, acarina, and actinopterygii were separated from each other. The amino acid sequence of *PcABCG23* showed the closest genetic distance to *ABCG23* of *T. urticae* while showing the farthest genetic distance to *ABCG23* of *Ictalurus punctatus*. Moreover, the genetic distances between acarina and insects were generally found to be close.

### 2.2. Expression of the PcABCG23

**Figure 4:**
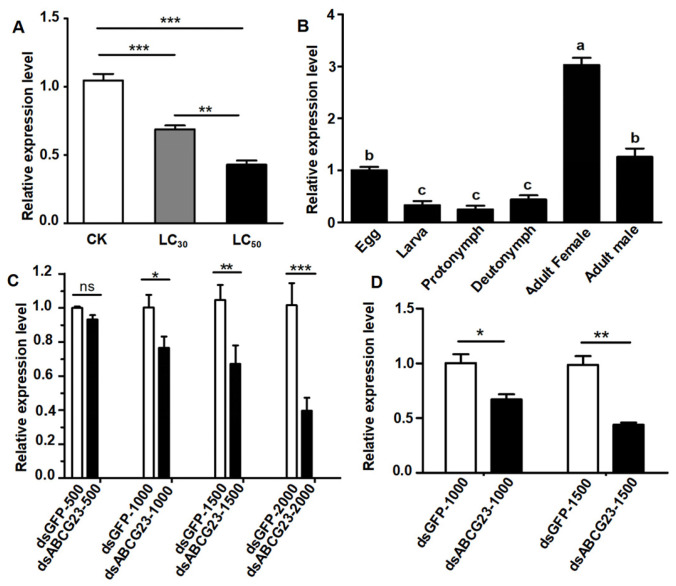
(**A**) mRNA expression levels of *PcABCG23* across different concentrations of spirobudiclofen stress, CK: (*n* = 200), LC_30_: (*n* = 200), LC_50_: (*n* = 200) were used for total RNA extraction. (**B**) mRNA expression level of *PcABCG23* across different developmental stages of *P. citri*. egg (*n* = 500), larva (*n* = 400), protonymph (*n* = 300), deutonymph (*n* = 300), adult females (*n* = 200), and adult males (*n* = 200) were used for total RNA extraction. Data represent the means ± SEM of three biological replicates. Different letters on the bars indicate significant differences among different samples (ANOVA, Tukey’s Honestly Significant Difference, *p* < 0.05). (**C**) Concentration-response of *PcABCG23* transcript to receive dsRNA (500 ng/μL, 1000 ng/μL, 1500 ng/μL and 2000 ng/μL with 50 μL ds*PcABCG23* 24 h); (**D**) RNAi efficiencies were tested 48 h after receiving 50 μLdsRNA (1000 ng/μL and 1500 ng/μL) ((ns) no significance, * *p* < 0.05, ** 0.001 < *p* < 0.05, *** *p* < 0.001).

Regarding developmental stages, the RT-qPCR results demonstrated that the expression of *ABCG23* was significantly higher in adults (both females and males) and eggs (Figure 4). Additionally, the expression of *ABCG23* was found to be higher in females than in males and higher in adults compared to eggs. However, there was no significant difference in the expression of *ABCG23* among the larva to deutonymph stages (Figure 4). According to different concentrations of spirobudiclofen stress, qPCR results showed a gradual decrease in the expression level of *ABCG23* as the concentration increased (Figure 4).

The transcriptional responses of *ABCG23* genes in *P. citri* adult females varied depending on the dsRNA concentration and feeding time (Figure 3 and Figure 4). The expression of *ABCG23* was significantly affected by dsRNA concentration and feeding time, except that the expression of *ABCG23* was not significantly affected by dsRNA fed to 500 ng/µL of dsRNA for 24 h (Figure 4). At all two feeding times, the expression of *ABCG23* was increased when fed dsRNA (Figure 4). However, the expression of ABCG23 was reduced by 37% when fed with 1000 ng/µL of dsRNA for 24 h and by 55% when fed with 1500 ng/µL of dsRNA for 48 h (Figure 4). RT-qPCR analysis indicated that the expression level of *ABCG23* was significantly lower in adult females fed on 1500 ng/µL of *ABCG23* dsRNA for 48 h than those fed with the control dsGFP.

### 2.3. Effect of PcABCG23 Knockdown in P. citri

**Figure 5:**
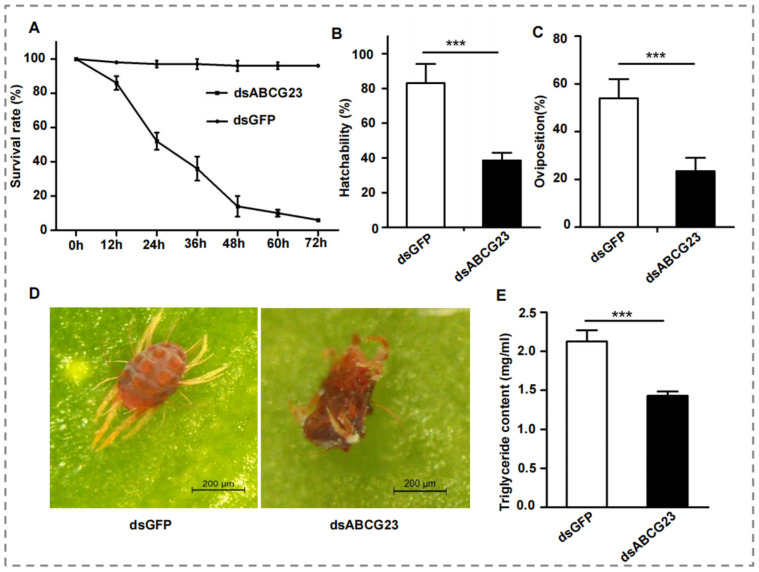
(**A**) The survival rate, (**B**) hatchability, and (**C**) oviposition of ds*ABCG23*-fed *P. citri* was recorded every 12 h; dsGFP-fed *P. citri* were used as the control (*n* = 60). Phenotypic images of adult females (**D**) and TG contents (**E**) of adult females from *P. citri* 48 h after being fed ds*ABCG23* and dsGFP. (Student’s *t*-test, *** *p* < 0.001).

The survival rate of adult females exhibited a significant decrease when *ABCG23* was silenced compared to being fed dsGFP for 48 h. Meanwhile, as the feeding time of dsRNA increased, the survival rate of adult females decreased more significantly, and the most significant decrease occurred at 24 h and 48 h (Figure 5). Moreover, the hatchability and oviposition of adult females were significantly lower when fed 1500 ng/µL of *ABCG23* dsRNA compared to being fed dsGFP for 48 h (Figure 5).

After the successful gene silencing of *PcABCG23*, we investigated whether the inhibition of mRNA expression of the *ABCG23* genes led to abnormal or lethal phenotypes. The *P. citri* phenotypes were observed and recorded at 48 h after *PcABCG23* administration, and phenotypic deformities were classified into body shrinkage and those that did not shed their skin normally (Figure 5). No such phenotype was seen in the dsGFP group. Overall, in the adult females after RNAi, individuals showed severe abnormal phenotypes, such as abnormal body shrinkage phenotypes, and small individuals who died because of an inability to molt were observed (Figure 5).

Because *ABCG23* showed a much lower expression in dsRNA treatment than dsGFP, which role in the synthesis of fatty acids was evaluated in *P. citri* using RNAi. Furthermore, treating adult females with *ABCG23* dsRNA resulted in a 39% decrease in TG contents compared to control groups treated with dsGFP (Figure 5). Therefore, silencing *ABCG23* led to a significant reduction in fatty acid synthesis in *P. citri*.

## 3. Materials and Methods

### 3.1. Mite Rearing and Acaricide

In 2019, a stable population of *P. citri* was established by collecting a colony from Nanchang University, Nanchang, China, and maintaining them through continuous breeding. *P. citri* were fed citrus tender leaves and kept in an artificial climate box (Percival Scientific, Model: ARC-36VL-DR, Haidian District, Beijing, China) with the following environmental conditions: a temperature of 25 ± 1 °C, a relative humidity (RH) of 70 ± 5%, and a photoperiod of 16:8 h. The population was not exposed to any pesticides until the present study (in 2022), which was considered the acaricide-susceptible strain (SS).

Spirobudiclofen at 24% SC, IUPAC: 3-(2,4-dichlorophenyl)-2-oxo-1-oxaspiro [4,5] dec-3-en-4-yl dibutyl carbonate, was bought from Zhejiang Yulong Biotechnology Co., Ltd. (Jiaxing, China).

### 3.2. Cloning and Sequencing the ABCG23 Gene

#### 3.2.1. RNA Extraction, cDNA Synthesis

The total RNA was isolated from 200 adult females of *P. citri* using Trizol according to the manufacturer’s instructions (Sangon Biotechnology, Shanghai, China). Three biological replicates were set for each treatment group in all experiments.

The first strand synthesis kit (PrimeScript™ II 1st Strand cDNA Synthesis Kit, Takara Bio, Dalian, China) was used to reverse cDNA.

The SMARTer™ RACE 5′/3′ kit (Takara Bio, Dalian, China) was used to build libraries of 3′ RACE and 5′ RACE. Reverse-transcribed cDNA was placed at −20 °C.

#### 3.2.2. Target Gene Fragment Cloning

Partial sequences of *PcABCG23* were retrieved from the NCBI website (http://www.ncbi.nlm.nih.gov/gorf/gorf.html accessed on 2 May 2022). The open reading frames (ORFs) of the target nucleotide sequences were found using the ORF Finder at the NCBI website (https://www.ncbi.nlm.nih.gov/orffinder accessed on 5 July 2022) and verified via PCR. For full-length cDNA cloning, the rapid amplification of the cDNA Ends (RACE) kit (Clontech, Dalian, China) was used. Specific primers (Table 1) were designed using Oligo 7.0 software based on the intermediate fragment of *PcABCG23*.

### 3.3. PcABCG23 Expression Analysis

After the eggs were produced, developmental stages, including the egg (*n* = 500), larva (*n* = 400), protonymph (*n* = 300), deutonymph (*n* = 300), adult females (*n* = 200), and males (*n* = 200), were collected to determine the transcription levels of *PcABCG23*.

Based on previous multi-omics studies [25], the experimental results for the toxicity determination of spirobudiclofen against *P. citri* were obtained, and two concentrations of spirobudiclofen (LC_30_ and LC_50_ treated groups) were used for the treatment. According to the leaf disc impregnation method, the adult females of *P. citri* were treated with two sublethal concentrations of spirobudiclofen (*n* = 500 and three biological replicates per treatment group). After 24 h, samples (*n* = 200, three biological replicates per treatment group) were collected for RNA extraction to determine *PcABCG23* expression levels.

The mRNA levels of the *ABCG23* gene were measured via RT-qPCR using the 2×TaqPCR Master Mix in a StepOne Plus real-time quantitative PCR system. Specific primer pairs for each gene were designed using Oligo 7.0 (Table 1). The qPCR cycling parameters were as follows: 95 °C for 10 min, followed by 40 cycles of 95 °C for 30 s and 60 °C for 30 s.

The RT-qPCR analysis was conducted as described [26], with the relative mRNA level of the target gene normalized to the expression of the internal marker genes glyceraldehyde phosphate dehydrogenase and elongation factor1a (*GAPDH* and *ELF1A*). The relative expression levels of *PcABCG23* were measured using three biological replicates (egg (*n* = 500), larva (*n* = 400), protonymph (*n* = 300), deutonymph (*n* = 300), adult females (*n* = 200), adult males (*n* = 200)) and each sample was calculated in triplicate using the 2^−ΔΔCT^ method [27]. The primers used for this analysis are listed in Table 1.

### 3.4. Synthesis of dsRNA and RNAi Efficiency Evaluation

#### 3.4.1. dsRNA Synthesis and Delivery

Additionally, the functional verification of the *ABCG23* gene with significantly different changes was performed via RNAi. RNAi experiments were subsequently performed using the collected adult females of *P. citri*.

Gene-specific primers (Table 1) containing a T7 polymerase promoter sequence were designed on the E-RNAi website (https://www.dkfz.de/signaling/e-rnai3/ accessed on 13 October 2022). The *Aequorea Victoria* (dsGFP) RNA sequence was used as a control. A T7 High Yield Transcription Kit (Vazyme Biotech Co., Ltd., Nanjing, China) was used for dsRNA synthesis. dsRNA was diluted with RNase-free water adjusted to the final concentration at 10 μg/μL and stored at −80 °C for RNAi experiments.

For the RNAi assay, the dsRNA sequences were introduced into *P. citri* through the feeding pattern. Adult females who were not exposed to insecticides were transferred to the citrus leaf dish (2 × 2 cm) with dsRNA for feeding. After that, the adult females were cultured in a climate-controlled chamber. After 24 h and 48 h of RNAi treatment, the samples were collected (*n* = 200, with 3 biological replicates per treatment) for RNA extraction.

#### 3.4.2. RNAi Efficiency Evaluation

To establish the optimal dsRNA concentration of *PcABCG23*, adult females were fed with 500, 1000, 1500, and 2000 ng/μL dsRNA, and qRT-PCR was used to determine RNAi efficiency after 24 h. Two optimal dsRNA concentrations of *PcABCG23* were identified, and RNAi efficiency was assessed at 24 h and 48 h after feeding to determine the duration of *PcABCG23* silencing. Each sample contained 50 dsRNA-fed *P. citri*, and each treatment contained 3 biological replicates.

### 3.5. Evaluation of Biological Changes in P. citri after RNAi

#### 3.5.1. Biological Observation after RNAi

We placed 60 adult females fed to dsRNA on citrus leaves to evaluate whether the *PcABCG23* had adverse effects on their development and reproduction. Before calculating the survival rate and fecundity, we observed the individuals every 12 h until they died. dsGFP-fed individuals served as the control group (3 biological replicates per treatment group).

#### 3.5.2. Evaluation of Fecundity after RNAi

To evaluate the fecundity of *P. citri* after *PcABCG23* silencing, the adult females (*n* = 60) were transferred to citrus leaves in a climate-controlled chamber. After the ds*ABCG23* and dsGFP were fed for 48 h, the oviposition, hatchability, and survival of individuals were counted every 24 h until death.

#### 3.5.3. Determination of Triacylglycerol (TG) after RNAi

To evaluate the TG contents of *P. citri* after *PcABCG23* silencing, 300 adult females were prepared for an RNAi assay, and 60 surviving *P. citri* adult females were collected for determination. The TG contents were measured using the double antibody sandwich method, following the insect TG test instructions of the ELISA kit (Jingmei Biotechnology Co., Ltd., Yancheng, China). The OD values were determined using a photometer set at 450 nm. The amount of TG in the sample solution was computed based on the standard curve (3 biological replicates per treatment group). The experiment was completed by Wuhan Seville Biological Co., Ltd., Wuhan, China.

### 3.6. Statistical Analysis

Data analysis was performed using one-way ANOVA and Student’s *t*-test. The data were presented as the mean ± standard error. To evaluate the differences in the expression level of *PcABCG23* across different stages and under two concentrations of spirobudiclofen stress, ANOVA followed by Tukey’s Honestly Significant Difference test was utilized. Student’s *t*-test was used to determine the statistical significance of the difference between the control and treatment groups.

## 4. Discussion

ABC transporters have been found to have a significant impact on insecticide resistance, as well as playing a crucial role in various physiological processes such as the construction of cuticle barriers, the synthesis of 20-hydroxyecdysone, transportation of xenobiotics, and the development of wings in aphids [6,10,28,29,30]. Previous studies have identified certain target genes, such as *FAS, ELO, FAR*, and *P450*, that are involved in the cuticular penetration barrier function, specifically related to lipid synthesis [31,32,33,34]. All animals, including insects, in all stages of their life cycle, necessitate the transportation of internal lipids from production sites to the cuticle and developing embryos [35]. Insects primarily rely on *ABCG23*, a major transport protein that acts as a reusable carrier, to accept and transport lipids between different tissues. At present, the involvement of *ABCG23* in cuticular lipid accumulation and its precise role in *P. citri* reproduction remains unclear.

Therefore, we focused on the *PcABCG23* gene and explored whether it contributes to the cuticular penetration barrier function. The *ABCG23*-coding gene was cloned and characterized based on *P. citri* genome sequences. Our results show that the *ABCG23* gene shares similar characteristics with other species, as indicated by its AAA domain (Figure 2 and Figure 3). However, we also observed certain differences in the number of transmembrane domains.

To explore the function of *PcABCG23*, we determined the development-dependent mRNA expression of *ABCG23* in *P. citri.* Our findings demonstrate that the transcript of *PcABCG23* was abundantly expressed during the egg and adult stages of *P. citri*, while it was poorly expressed in other stages (Figure 4). This study suggests that the active expression of *PcABCG23* can impact the reproduction of *P. citri*. Previous research has shown that insect eggs require an adequate nutrient reserve for successful embryonic development [36]. During oogenesis, lipids serve as the primary energy source for embryo development and must accumulate in growing oocytes. This is especially significant as lipids provide almost all the energy required for developing oocytes in mosquitos, such as *Culex quinquefasciatus* [37]. It can promote egg development by providing sufficient energy, which makes the lipid transport function particularly important.

To investigate the transcriptional response of *PcABCG23* to spirobudiclofen stress, this study examined the mRNA expression in two sublethal concentrations of spirobudiclofen-treated groups of *P. citri*. The results show that the mRNA expression level of *ABCG23* decreased with an increasing insecticide concentration. Our findings suggest that simply observing changes in transcript levels in response to insecticide treatment is not enough to determine if a particular gene is involved in insecticide detoxification. Numerous transcriptomic studies have reported changes in gene expression in response to insecticide treatment at sublethal concentrations across various insect species, including *Bactrocera dorsalis*, *Drosophila melanogaster*, and *Plutella xyostella* [38,39,40,41]. According to these studies, a large number of genes were determined to be changed in response to insecticide stress. However, it seems unlikely that all of these genes are directly involved in pesticide elimination. These findings are important for pest management as even low concentrations of pesticides may not exhibit a clear phenotypic change, and the regulation of genes affecting the entire genome can have unknown physiological and ecological effects.

In this work, we were particularly interested in *ABCG23*, which is involved in barrier formation and function. Insect epidermal lipids are mainly found on the surface of the cuticle. They help prevent the penetration of exogenous and potentially harmful substances [42]. Their amounts and composition include CHCs, sterols, fatty alcohols, triglycerides, fatty acids, and wax esters that vary by stage and species [21].

To analyze the possible biological role of *PcABCG23* in the epidermal lipid transport and reproduction of *P. citri*, this gene was silenced using RNAi technology to observe the specific phenotype of *P. citri* adult females. An RNAi phenotype was found during the silencing of *PcABCG23*, which resulted in a significant decrease in the oviposition, hatchability, and survival rate. Overall, this phenotype was described for the knockdown of the *ABCG23* gene as *P. citri* was not able to shed the old cuticle. Moreover, these adult females had wrinkles upon observation. Notably, the content of triglyceride significantly decreased when the *ABCG23* gene was silenced. The phenotype of wrinkling is similar to the previously described phenotype of knocking down the lipoprotein (*Lp*) and lipoprotein receptor (*LpR*) in *Locusta migratoria* [21]. At the same time, we also found a phenomenon of pigment deposition in *P. citri*, also known as the melanization reaction. However, the blackening reaction is an important and special innate immune defense mechanism in insects. Among them, melanin is an important component of an insect’s sturdy epidermis, playing a barrier-protective role. As far as we know, this is the first study to verify the role of *ABCG23* in cuticle lipid transportation and the reproduction of *P. citri.*

Here, our results indicate that the *ABCG23* gene does not function alone but together with other mechanisms to promote the synthesis of cuticles. Further work is needed to assess the contribution of *ABCG23*. In summary, the *ABCG23* transporter gene was suggested to be involved in cuticle lipid transportation and the reproduction of *P. citri.* The results help to better understand the evolution of cuticles and further study the function of the *ABCG* gene in cuticle lipid synthesis (Supplementary Materials).

## Figures and Tables

**Table 1:** Primers for PCR, dsRNA and RT-qPCR.

	Primer Sequence (5′-3′)
For gene cloning	
*PcABCG23*-F	TTTCGTCACCGATCCAGTCC
*PcABCG23*-R	TGACACGACTTAGGGCGAAC
*PcABCG23-3*′F1	TCGTGTCCGCTCTCTGTGA
*PcABCG23*-3′F2	CTTATTTCCGTTGGCTGTG
*PcABCG23*-5′R1	CGAAGTGCTGCTGATGGTGAT
*PcABCG23*-5′R2	TGCTGCTGATGGTGATTGC
UPM long	CTAATACGACTCACTATAGGGCAAGCAGTGGTATCAACGCAGAGT
UPM short	CTAATACGACTCACTATAGGGC
NUP	AAGCAGTGGTAACAACGCAGAGT
For qRT-PCR	
* PcGAPDH * -F	CTTTGGCCAAGGTCATCAAT
* PcGAPDH * -R	CGGTAGCGGCAGGTATAATG
* PcELF1A * -F	GGCACTTCGTCTTCCACTTC
* PcELF1A * -R	ATGATTCGTGGTGCATCTCA
*PcABCG23*-qF	TATGGAATGGTCGTGTCCGC
*PcABCG23*-qR	CGCGTATTCACACAGCCAAC
For *ABCG23* dsRNA synthesis	
T7 GFP ds-F	TAATACGACTCACTATAGGGCCACAAGTTCAGCGTGTCCG
T7 GFP ds-R	TAATACGACTCACTATAGGGTGGGTGCTCAGGTAGTGGTTGT
T7 *ABCG23* ds-F	TAATACGACTCACTATAGGGCGGTTGTCTTCGACCTCAAT
T7 *ABCG23* ds-R	TAATACGACTCACTATAGGGCTTGTCTCATGAAGCCCACA

## Data Availability

Data is contained at: https://www.alipan.com/s/aXVE9BxM2Xz (accessed on 1 March 2023).

## Supplementary Materials

The following supporting information can be downloaded at: https://www.mdpi.com/article/10.3390/ijms25020827/s1.
