# Supplementary material for: Knockdown of the ABCG23 Gene Disrupts the Development and Lipid Accumulation of Panonychus citri (Acari/Tetranychidae)
Source: Int J Mol Sci. 2024 Jan 9;25(2):827. doi: 10.3390/ijms25020827 (PMC10815512; doi:10.3390/ijms25020827)
Supplement: Supplementary file 1 [file ijms-25-00827-s001.zip › ijms-2732762-supplementary.pdf]

>ABCG23
ACATGGGGAGACGTGGTGAAGCGTTTCTCAAGTCAAAACCTTTTTACTTTCCTTTTTACC
TTTAGAGAGTAGTGATTTCAACACCATCTTCCTTACCTTTTCCTCTAGATTGTTTGATTA
ACAAAAGTGACCTGAGGATCGCCAATTTAACTTGAAATCTTTTTAACTTTTTTTACTTGT
TGAATCTTCATCAGCCCCGAAATGGGTGCACCAAAAGATTTAACCGTGTGTAGCAACAAT
TCGGTAAAAATATCGGTTCCGTGGCAATCACCATCAGCAGCACTTCGTCAACAGTTATCA
ACTTCATCATCGTTGTACAATCATCTTCATCACTCAACCACCAACAATTCCAATTCTTCG
TCTTCTCCTGCTACAAATGGACTTGTTATTCGTGGTTTAGGTTATTTGACCAAAAAGAAG
GAGGAAATTCTGAGGGACATCAACATGACGGTTCCAAAAGGTACAATTTACGGCCTATTG
GGACCCAGTGGATGTGGTAAAACAACTTTACTTCGTTGTGTCGTCGGTTGTCTTCGACCT
CAATATGGACAAATCATGATTTTTGGTAAACAGCCAGGCCGTGAAACCGCTCAAATTCCT
GGTACTGGTGTGGGCTATATGCCTCAAGAAAATACGCTCTATGAGAATATGACCATAGAA
GAAGTTTTAATCTATTATGGTCGAGTATTTTTTCTATCGATTGAAGAGCTTTATGAAAGA
ATCAAAGGATTAATGGGAGTTCTTGAACTTCCGGATAAAGGTCGAATGATCAAAAAGCTC
AGTGGGGGACAAAAGCGACGTGTTTCTTTCGCTATCGCTCTCATTCATCGACCAGGTCTC
TTGATTCTAGATGAACCTACTTCAGGAGTCGATCCTTTATTGAGGGAAAAACTTTGGCAA
CATTTAATGTGCATTTCTAAAAATCAAGGAGCCACAATAATCATAACAACCCATTACATT
GAAGAGGCTCGCAGGTCACATAATGTGGGCTTCATGAGACAAGGAAGACTTTTAATGCAA
GAAGATCCTGATCGCCTTTTAGCAGATCGTGGGGTCGAAACACTGGAAGATGCTTTCCTC
GATATCTGTAGGAATCAAGGTAGAATCAGTTGTGTTCCTGAACAAGAAAAGATAGAGTAC
AAGGGAAATGTTCCAACTGCAACTTTCGTCACCGATCCAGTCCTGATTAAGAAACGAATC
CGCGAATGGTTTTTCATATTCTTTGGTGCCCTTTGGCGACATTTAGTCAGTGATATTCGA
GAACCTTTGACTATTGGCTTTCAATACGTCATTCCAATTCTGTCCATGTTACTGTTTGCC
CTTTGTATCGGTGGTCAACCATTTGACATTCCTCTTGGAATTGTGAACGAAGAAGTTGGA
TTTGAAGGATTTTTGGGTCGACAGTTTGTTGGCTCTTTGGATCCTCATCTTTTCAATGTT
TTCAACTATTCGGATCTTAGTGAGGCAGCTGAAGCGGCCAAAGTTCGTGAAGTTTGGGGA
TATCTCCATATAAAAAGAGGATTCACTGAATCGCTATACGATGTTCTGTTTCCCGATAGT
GAACCACTTAATTCATCAATCCAATCAAGTCATATCAACCTCCATGCCGATCTAACAGAT
CGAGTAATCACAACTACGATGCATCGCTATTTAGATGAGTCTTTTATCAGTTTTGTGCAA
CAGGTTATTAAAGATTACAACAAAGAGGATCCTGATGTCC GAATGCCACAATTTATCGCC
AATCTTCCAATCAAAATGGGATCACCGATCTATGGACGATTAGAGAAGAAAAGTTATCGT
GGTTATCGTGATTTCATGTTGCCAGGATTAATTGTCAACATAACTTATGCCATTGCCTAT
TCTCTTACAGCACTTAATCTAATTAATGAGCGTAAAAATCAAACTTTCGAGAGAAATTAT
GTCGCTGGTGTTAAACCTTCGCAAATGTTATTAGCGTTCG CCCTAAGTCGTGTCATCATG
ATGTCCCTTTATCTCTTCTTGATCATTTATCTTCCCATCGCTGTCTTCAG TATGCCAGTC
GATTGTATTCATATTCTTCATTCACTTCCACTTTTGATGC TACTCAATATCTCTGGAATG
ACCTATGGAATGGTCGTGTCCGCTCTCTGTGATTCAATTGAACAATGTGCCGTTTTCTCG
GCTGCCACTTTATTTATAATTCTTTTCATGAGTGGAACAATTTGGCCTATGGAAGCAATT
CCAGCTTATTTCCGTTGGCTGTGTGAATACGCGCCGACAMCGGACGGCCAATGAAGCCCT
GCGTGATATTTGGTATAAAGATTGGAGTATTTTCTCACCGCGAGTGGCCACCGCTCACGG
AATTACACTTGTCTGGACATTTGTTTTCTTTTTAATTGGTTTAAGATTTTTTAGACTAAG
CAAATAATTAACTTTCAATTTTTACAAACCATTTTCTTGTAAATCATCATCAATCTAACC
ACCATCGATAATTTCATCAATCACCATCATTTTTATCACTTTTATATTTTAATACTTTTT
GTAAAACCCAAATGCCAAAGAAAACCTTTTTTTGCATATTCATTGAGCGCAAAAAAATGA

TCAACAATCAACACAAACACACAAACACGTATAAAGAAATTAAAATATGTATTTTTTATA
TTTATGATTTTAAAATCAAGAAAATAGATTAAAAAAGTTGAGAATTAATCAGTTTTGAAA
TTAAAAAATATAAACGAAAACACATAAAAAAAAAAAAAAAAAAAAAAAAA
